# Decreased Reactive Oxygen Species Production in Cells with Mitochondrial Haplogroups Associated with Longevity

**DOI:** 10.1371/journal.pone.0046473

**Published:** 2012-10-29

**Authors:** Ai Chen, Nicola Raule, Anne Chomyn, Giuseppe Attardi

**Affiliations:** Division of Biology, California Institute of Technology, Pasadena, California, United States of America; UMASS-Amherst/Tufts University School of Medicine, United States of America

## Abstract

Mitochondrial DNA (mtDNA) is highly polymorphic, and its variations in humans may contribute to individual differences in function. Zhang and colleagues found a strikingly higher frequency of a C150T transition in the D-loop of mtDNA from centenarians and twins of an Italian population, and also demonstrated that this base substitution causes a remodeling of the mtDNA 151 replication origin in human leukocytes and fibroblasts [1]. The C150T transition is a polymorphism associated with several haplogroups. To determine whether haplogroups that carry the C150T transition display any phenotype that may be advantageous for longevity, we analyzed cybrids carrying or not the C150T transition. These cybrids were obtained by fusing cytoplasts derived from human fibroblasts with human mtDNA-less cells (ρ^0^ cells). We chose for cybrid construction and analysis haplogroup-matched pairs of fibroblast strains containing or not the C150T transition. In particular, we used, as one pair of mtDNA donors, a fibroblast strain of the U3a haplogroup, carrying the C150T transition and a strain of the U-K2 haplogroup, without the C150T transition, and as another pair, fibroblasts of the J2b haplogroup, carrying the C150T transition and of the J1c haplogroup, without the C150T transition. We have found no association of respiratory capacity, mtDNA level, mitochondrial gene expression level, or growth rate with the presence of the C150T transition. However, we have found that the cybrids with haplogroups that include the C150T transition have in common a lower reactive oxygen species (ROS) production rate than the haplogroup-matched cybrids without that transition. Thus, the lower ROS production rate may be a factor in the increased longevity associated with the U and the J2 haplogroups. Of further interest, we found that cybrids with the U3a haplogroup exhibited a higher respiration rate than the other cybrids examined.

## Introduction

A C150T transition in mitochondrial DNA (mtDNA) was found to occur more frequently in centenarians and in twins of an Italian population [1]. The C150T base substitution, which is located in the mtDNA D-loop region and causes a remodeling of the mtDNA 151 replication origin in leukocytes, was found to be homoplasmic in about half of the leukocyte samples in which the base substitution was observed. Because of this homoplasmy and because the C150T transition is a commonly occurring polymorphism, it is likely in these cases that the base substitution is an inherited polymorphism rather than a somatically acquired mutation.

In fact, the C150T polymorphism is associated with several haplogroups or subhaplogroups, including J2, D5, M7b, T2, U3, U5, and N9a [2]. Furthermore, observations of associations between haplogroup and longevity have been reported. For instance, DeBenedictis and colleagues found a higher frequency of the J haplogroup in healthy older men from northern Italy [3]. Similarly, Niemi and colleagues found that the haplogroup frequencies in a sampling of very old individuals (vitality 90+) in Finland differed from those in the middle-aged controls, haplogroups U, K (U-K) and J being more prevalent among the old individuals [4]. The subhaplogroups of U were unspecified in that study. More recently, in Finnish and Japanese subjects, 150T and two additional common polymorphisms, 10398G and 489C, all of three of which occur in the J2, D5, and M7b haplogroups, were associated with longevity [5]. The C150T transition was not associated with longevity in the U5, T2, and N9a haplogroups. Relevant to the work we present here, the J2 haplogroup, with T at position 150, was found at a higher frequency, and the J1 haplogroup, with C at position 150, at a lower frequency, in very old individuals than in a control population [4]. Besides the non-coding region C150T polymorphism, longevity has also been found to be associated with mtDNA coding region polymorphisms such as 5178A, which defines haplogroup D, occurring in Asian individuals, and 9055A, which defines haplogroup K [6] [7], [8]. However, conflicting associations with regard to the 5178A polymorphism have been reported [9]. The aforementioned associations are based on population genetics. The consequences on mitochondrial function of these longevity-associated polymorphisms, if any, are unknown. At present there have been few studies that identify functional effects of DNA polymorphisms [10]–[13].

In order to understand the effect of the C150T transition or of its associated haplogroup polymorphisms, we have analyzed cybrids obtained by fusing cytoplasts derived from human fibroblasts carrying or not the C150T transition with human mtDNA-less cells (ρ^0^ cells) derived from an osteosarcoma cell line [14]. In particular, we found, among the fibroblast strains used in the previous study by Zhang and colleagues [1], two pairs that were matched in haplogroup but differed at position 150 (Table 1).

**Table 1:** Fibroblast strains and their haplogroups.

Fibroblast	Name in	nucleotide	Haplogroup	Cybrids
strain	Zhang *et al*., 2003	at pos. 150		
AG07309	LS11-1	T	U3a	TF3A5
				TF7G11
				TF2D9
				TF4B2
				TF5A12
				TF11H2
AG13152	LS4-2	C	U-K2	E8
				H5
				G11
				H9
				T8
				E5
AG07135	LS9-1	T	J2b	F8
				F1
				IP2
				IP1
				2P1
				B3
AG14421	LS10-2	C	J1c	TFA24
				TFA15
				TFA7
				TFA12
				TFA16

The first pair was of the U haplogroup. One fibroblast strain was of the U3a subhaplogroup and carried the C150T transition. The other fibroblasts were of the U-K2 subhaplogroup and had C at position 150. The other pair of fibroblasts strains was of the J haplogroup. The members of this pair were of the J2b (C150T) and J1c (150C) subhaplogroups. Heretofore we will refer to subhaplogroups as haplogroups. These fibroblast strains served as mtDNA donors in the construction of cybrids. The use of cybrids made with the ρ^0^ cell line allows us to observe the effects of mitochondrial polymorphisms without the confounding effects of the varying nuclear backgrounds.

We have searched for phenotype differences between cybrids of different haplogroups. We have analyzed growth rate, respiratory rate, mitochondrial protein synthesis rate, mtDNA level, steady state level of components of the respiratory chain and reactive oxygen species (ROS) production rate. We have found that the one parameter that correlates with the presence of the C150T transition is a lower ROS production rate. ROS have long been thought to play a role in aging [15]. Thus, longevity that is associated with particular haplogroups may be at least partly explained by the relative level of ROS production that is allowed or specified by those haplogroups.

## Materials and Methods

### Cell line and culture conditions

The following human fibroblast strains, obtained from the American Type Culture Collection and listed in Table 1, were used as donor cells for introducing mitochondria and mtDNA into mtDNA- less (ρ^0^) cells: AG07135 (150T, LS9-1 in Michikawa *et al.* [16]), AG14421 (150C, LS10-2), AG07309 (150T, LS11-1), AG13152 (150C, LS4-2).

The fibroblasts were grown in high glucose Dulbecco's modified Eagle's Medium (DMEM; containing pyruvate, Gibco), supplemented with 10% fetal bovine serum (FBS). The mtDNA-less ρ^0^ 143B.206 cell line [14], derived from the osteosarcoma cell line 143B.TK^−^, was grown in DMEM supplemented with 10% FBS and 50 µg of uridine per ml. All cybrid cell lines were maintained in the same medium as the fibroblast cell lines.

### Mitochondria-mediated transformation

Transformation of mtDNA-less ρ^0^ 206 cells was carried out as described by King and Attardi [14], by fusing human fibroblast cells, which had been enucleated by centrifugation in the presence of cytochalasin B, with ρ^0^ 206 cells in the presence of 40% polyethylene glycol 1500 (PEG, BDH). Mitochondrial transformants were isolated in medium lacking pyruvate and uridine, specifically DMEM without pyruvate supplemented with 5% dialyzed FBS. 100 µg/ml BrdU was included in the medium to select against hybrids and unenucleated donor cells and for cybrids carrying the thymidine kinase deficiency marker of the ρ^0^ nucleus.

### Growth measurements

All cybrid cell lines were grown in DMEM medium with 10% FBS for seven days before the growth rate experiment was done. Cells were then plated on multiple 10-cm plates at 10^5^ per plate and were counted daily for 7 days. The population doubling time (DT) of the cell lines in DMEM medium, supplemented with 10% dialyzed FBS, was determined from growth curves or by using the formula: DT = (*t* – *t*_0_)log2/(log*N* – log*N*_0_), where *t* and *t*_0_ are the times at which the cells were counted, and *N* and *N*_0_ are the cell numbers at times *t* and *t*_0_, respectively.

### Analysis of mitochondrial protein synthesis

Pulse-labeling of the cell lines for 45 min with [^35^S]methionine–[^35^S]cysteine (ExPress ^35^S^35^S, Perkin Elmer) in methionine-free DMEM in the presence of 100 µg/ml emetine and electrophoretic analysis of the translation products were carried out as detailed previously [17]. The dried gel was exposed to a PhosphorImager screen. Quantification of radioactivity in the entire lane of the gel or in individual well-resolved bands was done using ImageQuant software.

### O_2_ consumption measurements

Rates of O_2_ consumption in intact cells at 37°C were measured polarographically by using a Clark-type oxygen electrode connected to a computer-operated Oxygraph control unit (Hansatech Instruments, Norfolk, England). 1∼2×10^6^ cells were suspended in 1 ml of Tris-based, Mg^2+^/Ca^2+^-deficient (TD) buffer (0.137 M NaCl, 5 mM KCl, 0.7 mM Na_2_HPO_4_, and 25 mM Tris-HCl [pH 7.4] at 25°C) for the analysis, as previously described [14]. Polarographic analysis of digitonin-permeabilized cells, using respiratory substrates and inhibitors to test the activity of the individual respiratory complexes, was carried out as detailed previously [18].

### Membrane potential ananlysis

Cells were stained with 10 nM tetramethylrhodamine ethylester perchlorate (TMRE, Molecular Probes) in DMEM without serum for 45 minutes. Then cells were harvested by trypsinization, resuspended in PBS with BSA (2.5 mg/ml) containing 10 nM TMRE and analyzed by flow cytometry in a FACSCalibur analyzer (BioRad).

### Determination of ROS generation

The intracellular ROS levels of cybrid cells were determined using CM-H_2_DCFDA (Molecular Probes). For these experiments, cells were collected and incubated with phosphate-buffered saline (PBS) and 1 µM CM-H_2_DCFDA. After a 30 min incubation period at 37°C, cells were centrifuged and resuspended in PBS. Measurements at 1 min intervals of the levels of fluorescence were begun immediately using Molecular Devices fluorescence plate reader and were continued for 1 h (excitation 485 nm and emission 538 nm). The rates of ROS generation were derived from the data.

### Mitochondrial DNA analysis

a. Allele-specific termination of primer extension for identifying cells with the C150T transition has been described previously [1]. Briefly, mtDNA fragment Init-Tra-Rep [16] was amplified from total cellular DNA. To detect the C150T polymorphism, primer extension was carried out with the primer 5′-GCAGTATCTGTCTTTGATTCCTGC CTC (positions 121–147 in the Cambridge sequence, [19], [20]) in the presence of dA, dT, and ddC.

b. Restriction fragment length polymorphism for determining mtDNA haplogroups. The mtDNA sequence was amplified by PCR using the primer pairs and amplification conditions described by Torroni et al. [21]. The PCR segments were digested with specific restriction endonucleases to distinguish haplogroups U, U-K, H, I, and J [21].

c. Total DNA sequencing: Total DNA from cybrid cell lines was extracted by using standard procedures. The complete mtDNA was amplified in 22 overlapping PCR fragments by the use of sets of the light-strand and the heavy strand oligonucleotide primers, listed in Table S1 [22]. Each PCR product was purified and subsequently submitted for sequence analysis. Sequencing was from M13 forward and M13 reverse primers, the sequences of which were incorporated in the PCR primers. The resultant sequence data were compared with the updated consensus Cambridge sequence of mtDNA (GenBank Accession No.: NC_012920) [20]. Identifications of polymorphisms as haplogroup- and subhaplogroup-specific are based on a published comprehensive phylogenetic tree [2]. The single nucleotide polymorphism database of MitoMap (http://www.mitomap.org) [23] and the comprehensive phylogenetic tree (http://www.phylotree.org) [2] were searched to determine whether the non-subhaplogroup polymorphisms that we had found had been reported previously.

### Statistical analysis

Except for the protein synthesis data, data are expressed as mean ± standard error of the mean. Statistical significance was determined by unpaired, 2-tailed t-test (http://graphpad.com/quickcalcs/ttest1.cfm).

## Results

### Generation of cybrid cell lines with different mtDNA haplogroups and growth properties

Fibroblast strains that were used by Zhang *et al.* [1] were subjected to haplogroup analysis [21]. Strains AG07309 (LS 11) and AG13152 (LS 4) were identified as belonging to Haplogroups U and U-K, respectively, and strains AG07135 (LS9) and AG14421 (LS 10) were identified as belonging to Haplogroup J. Subsequent DNA sequence analysis of the cybrids refined the haplogroup identities to U3a, U-K2, J2b, and J1c, respectively (Table 1). These four fibroblast strains were enucleated and subsequently fused to mtDNA-less human ρ^0^ cells (see Materials and methods). The cybrid clones derived from each fibroblast strain are listed in Table 1. We chose three or more transmitochondrial cybrid clones from each fusion for analysis. The presence or absence of the C150T transition in each clone was verified by allele-specific termination of primer extension [1]. Later, DNA sequencing of the mtDNA of several of the clones confirmed the base at position 150. Haplotype-matched C150T transition and “wild type” 150C clones were then used for the biochemical characterization described below.

**Figure 1:**
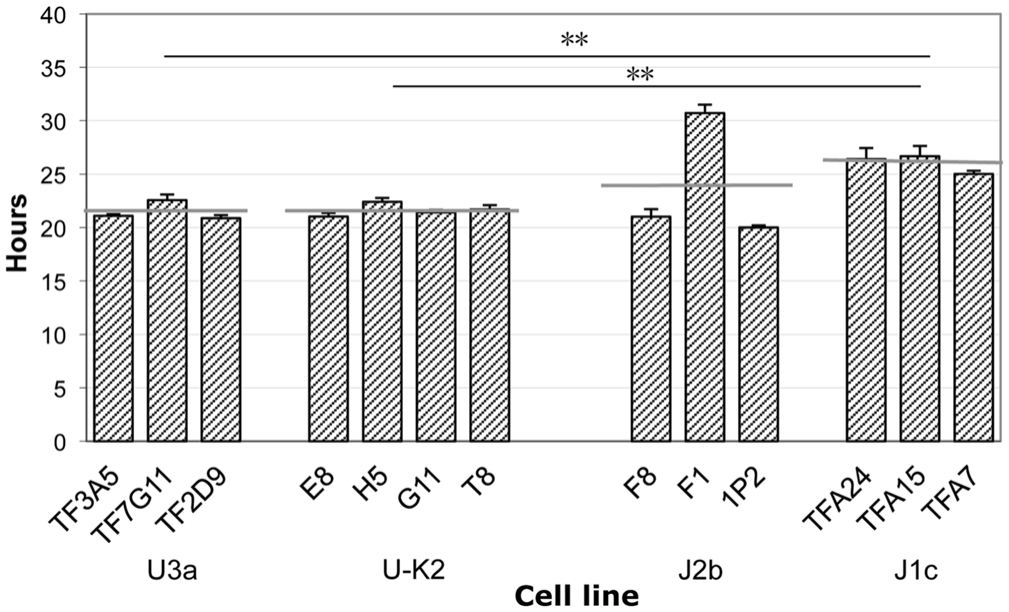
Growth rates of cybrid cell lines. The population doubling times during 7 days of growth are indicated. The cell lines are grouped by mtDNA donor cell and the haplogroup of the donor is indicated, namely U3a, U-K2, J2b, and J1c. The horizontal gray lines indicate the mean doubling time for each group of cybrids. A black horizontal line indicates that the difference between the doubling times of the cybrid groups at the ends of the line is significant. ** indicates P≤0.01, by the t-test.

Figure 1 shows that the cybrid cell lines of the two haplogroups U3a and U-K2 have a nearly identical doubling time, or growth rate. Also, whereas the overall mean doubling time of J1c is greater than that of J2b, the difference is not statistically significant.

### Mitochondrial protein synthesis and COXII protein level difference in two haplogroup pairs

**Figure 2:**
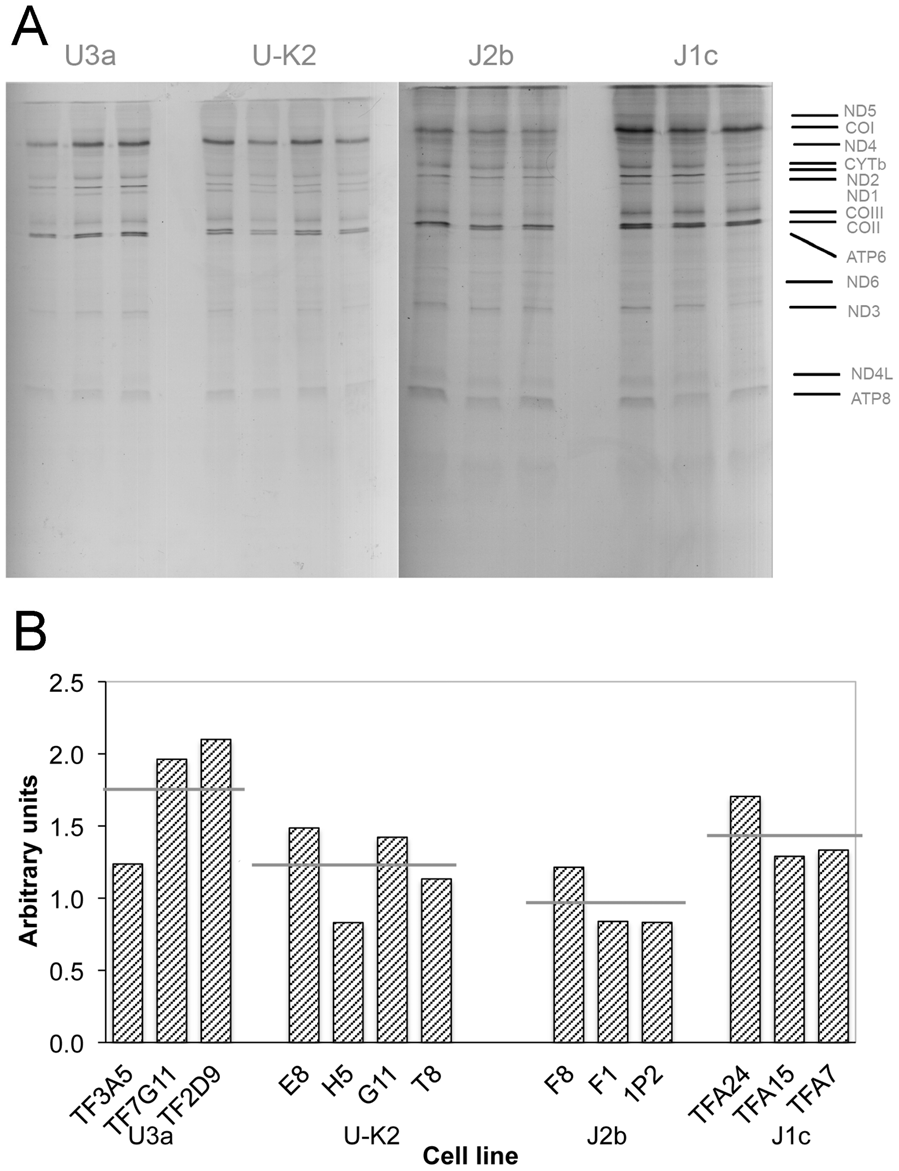
Mitochondrial protein synthesis rates. **A**, Electrophoretic patterns of the mitochondrial translation products of cybrid clones labeled for 45 min with [^35^S]methionine in the presence of 100 µg/ml of emetine. Each lane represents a different cybrid clone, the name of which is given below the corresponding bar in panel **B**. ND1, -2, -3, -4, -4L, -5, and -6, NADH dehydrogenase subunits 1, 2, 3, 4, 4L, 5, and 6, respectively; CYTb, apocytochrome *b*; COI, -II, and -III, subunits I, II, and III, respectively of cytochrome *c* oxidase; A6 and A8, subunits 6 and 8, respectively, of the H^+^-ATPase. **B**, Quantification of the labeling of the mitochondrial translation products shown in panel **A**. The cell lines are grouped by haplogroup, namely U3a, U-K2, J2b, and J1c. The horizontal gray lines in panel **B** indicate the mean level of labeling for each group of cybrids. Neither the difference between the U3a and the U-K2 cybrids nor the difference between the J2b and the J1c cybrids is statistically significant.

Figure 2**A** shows electrophoretic patterns of the mitochondrial translation products of the cybrids. Patterns of the mtDNA-encoded polypeptides of the 150T-carrying cybrids were qualitatively identical, in terms of electrophoretic mobility of the various polypeptides, to those of the 150C-carrying cybrid cells. Figure 2**B** shows a quantification of the results of the labeling experiments. The mean rate of labeling of the mitochondrial translation products in U3a (C150T) cybrids was higher than the mean value measured in the U-K2 (150C) cybrid cell lines. In contrast, the labeling rate in J2b (C150T) cybrids was lower than that in the J1c (150C) cybrid cell lines. However, because of the variability among the cybrid clones, the difference between the U3a and the U-K2 cybrids was not significant; nor was the difference between the J2b and the J1c cybrids. In addition, Western blots revealed that the variations in the steady state levels of complex IV subunit II (COXII) among the individual cybrids reflected, for the most part, corresponding variations in the overall rate of labeling of mitochondrial translation products (data not shown).

### Respiration differences between two haplogroup pairs

**Figure 3:**
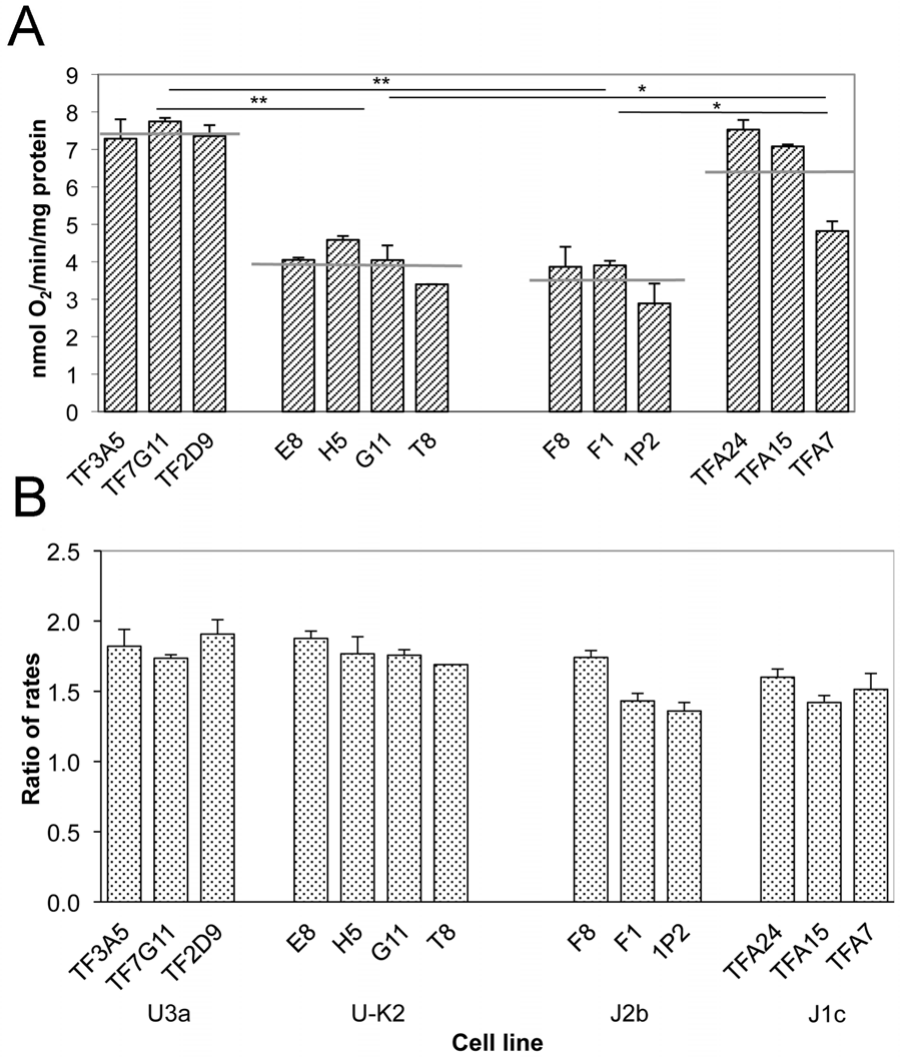
Respiration rates. **A**, Average rates of O_2_ consumption in intact cells, normalized to protein concentration, in individual cybrid clones are shown. Cell lines are grouped by haplogroup. A total of 3–6 determinations were made on each of three or four cybrid clones of each haplogroup. The error bars indicate the standard error of the mean. The horizontal gray lines indicate the mean respiration rate for the haplogroup. The horizontal black bars with asterisk indicate that the difference between the indicated groups is statistically significant; *, P≤0.05; **, P<0.01. **B**, DNP-uncoupled endogenous oxygen consumption rates, normalized to protein concentration, were measured 3–6 times for each cybrid clone. The ratios of uncoupled to endogenous respiration rates are shown.

The respiration rate of intact cells utilizing endogenous substrates was measured by polarography. As shown in Figure 3**A**, the U3a (C150T) cybrids exhibited higher rates of total O_2_ consumption, by 85%, relative to the U-K2 (150C) cybrids. In contrast, the J2b (C150T) cybrids showed a lower level of overall respiratory capacity, by 45%, as compared to the mean value of that of the J1c (150C) cybrids. The effect of the uncoupler dinitrophenol (DNP) on endogenous respiration, as revealed by the ratios of uncoupled to native respiration, was similar in U3a and U-K2 cybrids and similar in J2b and J1c cybrids (Figure 3**B**). Thus, the differences in endogenous respiration rates are not due to differences in the level of proton leakage of the inner membrane.

**Figure 4:**
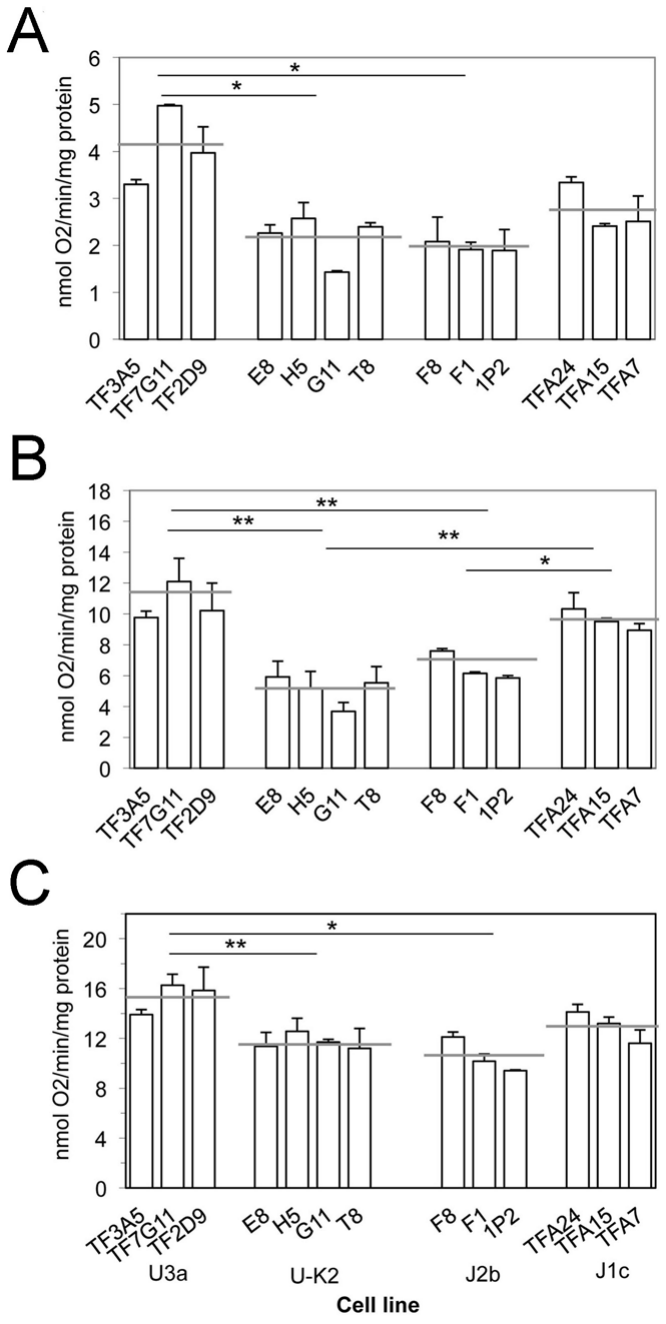
Analysis of O_2_ consumption in digitonin-permeabilized cells of the cybrid clones using different substrates and inhibitors. The activities of the various components of the respiratory chain were investigated by measuring the respiration rate dependent on (**A**) malate plus glutamate, on (**B**) succinate plus G3P (in the presence of rotenone) and on (**C**) TMPD plus ascorbate (in the presence of antimycin). Cell lines are grouped by haplogroup. A total of 3–4 determinations were made on each of the three or four cybrid clones derived from each fibroblast strain. The mean of those determinations is shown. The error bars indicate the standard error of the mean. Horizontal gray lines represent the average for each haplogroup. Horizontal black bars with asterisks indicate differences between averages are statistically significant; *, P≤0.05; **, P<0.01. P = 0.0578 for the difference between the means of respiration rate of the J2b and the J1c cybrids in panel **A**, i.e. not quite statistically significant.

In order to investigate differences in individual enzyme complexes of the respiratory chain, O_2_ consumption measurements were carried out on digitonin-permeabilized cells, using different substrates and inhibitors. As illustrated in Figure 4**A**, in the U3a (C150T) cybrids, the rate of malate/glutamate-driven respiration, which reflects the activity of complex I, was significantly higher, by 89%, than the average rate in the U-K2 (150C) cybrid cells. Similarly, the rate of succinate/glycerol-3-phosphate (G3P)-driven respiration, which reflects the activity of complex III, was significantly higher in U3a cybrids, by 110%, than the average rate in the U-K2 cybrids (Fig. 4**B**). Furthermore, the rate of *N*,*N*,*N*′,*N*′-tetramethyl-*p*-phenylenediamine (TMPD)/ascorbate-driven respiration, which reflects the maximum activity of Complex IV, was higher by 31% in U3a cybrids (Fig. 4**C**). J haplogroup cybrids showed the opposite trend; i.e., the J2b (C150T) cybrids showed generally lower rates than the J1c (150C) cybrids, i.e lower by ∼30% for malate/glutamate-stimulated respiration; by ∼30% for succinate/G3P-stimulated respiration; and by ∼20% for TMPD/ascorbate-stimulated respiration (Fig. 4). The relative rates of malate/glutamate-driven respiration among the different haplogroups paralleled the rates of respiration observed in intact cells, as expected.

### Membrane potential differences in two haplogroup pairs

**Figure 5:**
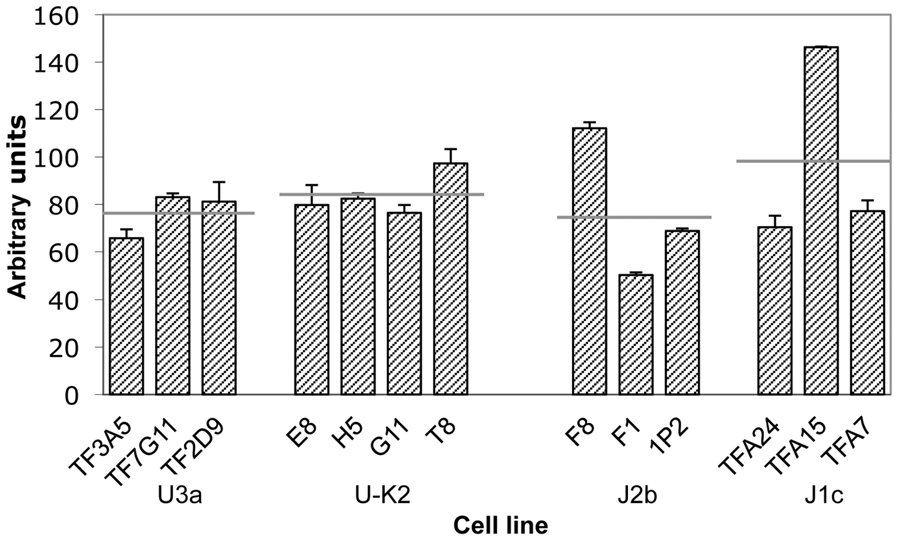
Membrane potential. Cybrid cell lines were stained with the fluorescent membrane potential indicator dye TMRE. The fluorescence was quantified by flow cytometry. A total of 2–3 determinations were made on each cybrid cell line and the mean values are shown. The error bars indicate the standard error of the mean. Gray bars represent the average for each haplogroup. Cell lines are grouped by haplogroup. No difference between any two groups of cybrids is statistically significant.

Maintenance of mitochondrial membrane potential (ΔΨ_m_) is necessary for production of energy (ATP), for protein import, and therefore, for the maintenance of most mitochondrial functions and the preservation of cellular homeostasis. Figure 5 shows the results of flow cytometric analysis in cybrid cells loaded with the ΔΨ_m_ indicator tetramethylrhodamine ethylester perchlorate (TMRE). The membrane potential is similar in U3a (C150T) cybrid cell lines and U-K2 (150C) cybrid cell lines. There was variability in the membrane potentials among the individual J2b (C150T) cybrid cell lines and J1c (150C) cybrid cell lines, with F8 and TFA15 cybrid cell lines showing a large deviation from the other cell lines within their respective haplogroups. The reason for this is not clear. The differences in membrane potential between haplogroups are not statistically significant.

### Common ROS generation tendency between two haplogroup pairs

**Figure 6:**
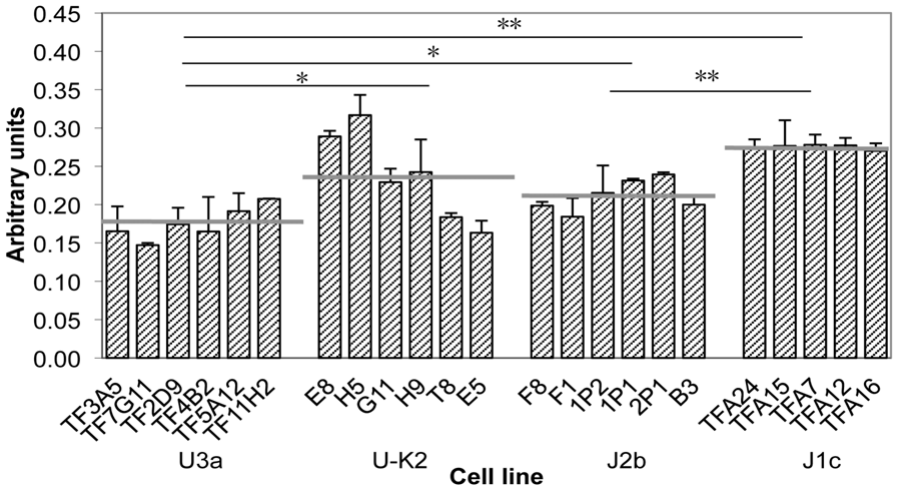
ROS generation rate. Cells of each cybrid clone were stained with the ROS-activated dye CM-H_2_DCFDA. Fluorescence data were collected on a Molecular Devices fluorescence plate reader for 1 h. A total of 2–5 rate determinations were made on each cybrid clone. Each bar represents the average fluorescence production rate for the indicated cybrid clone. The error bars indicate the standard error of the mean. The haplogroup is indicated below each group of cybrid clones. Horizontal gray lines represent the average for each haplogroup. Horizontal black bars with asterisks indicate differences between averages are statistically significant, as determined by a two-tailed unpaired t-test; *, P≤0.05; **, P<0.01.

The analysis of ROS production showed that the mean ROS production rate of the U3a (C150T) cybrid cell lines was 26% lower than that of the U-K2 (150C) cybrid cell lines (P = 0.0356) (and lower than those of the J2b and J1c cybrid cell lines (by 17%, P = 0.0142, and by 36%, P<0.0001, respectively)) (Figure 6). Similarly, the mean ROS production rate of the J2b (C150T) cybrid cell lines was 23% lower than that of J1c (150C) cybrid cell lines (P<0.0001). Because ROS production rate differences may arise from differences in ROS generation rate or differences in natural ROS defenses, we investigated whether the expression of ROS defense enzymes also differ. The MnSOD protein level, as revealed by Western blotting, did not differ between the U3a and the U-K2 cybrids, nor did it differ between the J2b and the J1c cybrids (data not shown).

### Mitochondrial DNA sequencing

We sequenced the entire mtDNA of TF3A5 (U3a), E8 (U-K2), T8 (U-K2), F8 (J2b) and TFA7 (J1c) cybrid cell lines. Overlapping fragments covering the entire 16.5-kb mtDNA were amplified from genomic DNA. We compared our sequences to the revised version of the Cambridge Reference Sequence. All the variants identified, except those that differ from the H2b- and individual-specific polymorphisms present in the Cambridge Reference Sequence, are reported in Tables 2, 3, and 4. The mitochondrial genome sequences of the haplogroup cybrid cell lines differed by 33–35 nucleotide substitutions from the revised Cambridge Reference Sequence. Some of the individual-specific polymorphisms are novel (Table 4). Only the TF3A5 cybrid cell line harbors heteroplasmic nucleotide changes, specifically in complex I subunits. The sequence of the U3a haplogroup cybrid revealed that almost all the coding region polymorphisms are U3a haplogroup specific; i.e., there are few individual specific polymorphisms aside from those that specify the U3a haplogroup.

**Table 2:** Control Region Polymorphisms in Cybrid Cell Lines.

TF3A5		E8, T8		F8		TFA7	
C150T	U3	T16519C	U8	T16126C	JT	T16126C	JT
A16343G	U3	T16224C	UK	C295T	J	C295T	J
G16390A	U3a	T16311C	UK	T489C	J	T489C	J
T16519C	U3a	T146C	K2	C16069T	J	C16069T	J
T16356C	U3a1c	T152C	K2a	C150T	J2	C462T	J1
C16301T		C64T		T152C	J2	G185A	J1c
				C16193T	J2b	G228A	J1c
						T482C	J1c1

The table lists all the Control Region polymorphisms found in the sequences of the mtDNAs of cybrids TF3A5, E8, T8, F8, and TFA7. The haplogroup and subhaplogroup with which the polymorphism is associated is indicated. Most of these Control Region polymorphisms are haplogroup-specific.

**Table 3:** Coding Region Polymorphisms, Haplogroup-specific.

TF3A5			E8, T8			F8			TFA7		
11467	syn ND4	U	11467	syn ND4	U	4216	**Y-H ND1**	R2′JT	4216	**Y-H ND1**	R2′JT
12308	tRNA Leu	U	12308	tRNA Leu	U	11251	syn ND4	JT	11251	syn ND4	JT
12372	syn ND5	U	12372	syn ND5	U	**15452**	**L-I CYTB**	JT	**15452**	**L-I CYTB**	JT
1811	A-G 16S	Ubr	1811	A-G 16S	Ubr	**10398**	**T-A ND3**	J	**10398**	**T-A ND3**	J
14139	syn ND5	U3				12612	syn ND5	J	12612	syn ND5	J
15454	syn CYTB	U3				**13708**	**A-T ND5**	J	**13708**	**A-T ND5**	J
4703	syn ND2	U3a				7476	tRNA ser	J2			
6518	syn COI	U3a				**15257**	**D-N CYTB**	J2			
9266	syn COIII	U3a				5633	tRNA ala	J2b			
**10506**	**T-A ND4L**	U3a				**15812**	**V-M CYTB**	J2b			
**13934**	**T-M ND5**	U3a				10172	syn ND3	J2b1			
2294	A-G 16S	U3a							3010	G-A 16S	J1
3010	G-A 16S	U3a1							**14798**	**F-L CYTB**	J1c
			9698	syn COIII	U8				**3394**	**Y-H ND1**	J1c1
			**9055**	**A-T ATP6**	UK/U8b				7184	syn COI	J1c1b
			14167	syn ND6	UK/U8b						
			3480	syn ND1	UK/U8b						
			10550	syn ND4L	UK						
			11299	syn ND4	UK						
			**14798**	**F-L CYTB**	UK						
			9716	syn COIII	K2						
			709	G-A 12S	K2a						
			**4561**	**V-A ND2**	K2a						

The table lists all the haplogroup- and subhaplogroup-specifying coding region polymorphisms found in the sequences of the mtDNAs of cybrids TF3A5, E8, T8, F8, and TFA7. The first column in each triplet indicates the nucleotide position of the polymorphism, the second column indicates the gene name and the nucleotide change (in the 16S or 12S rRNA genes) or the amino acid change or syn for synonymous substitution (in the protein coding genes), and the third column indicates the haplogroup or subhaplogroup with which each particular polymorphism is associated. Polymorphisms that cause an amino acid substitution are in boldface.

**Table 4:** Individual-specific polymorphisms.

TF3A5		E8, T8		F8		TFA7	
**3644**	**V-V+A ND1**	*1697*	*A-G 16S*	8911	L1-L2 ATP6	1966	G-A 16S
**4924**	**S-S+N ND2**			10454	tRNA arg	2623	A-G 16S
**10704**	**V-V+I ND4L**			*993*	*delA 12S*	***3862***	***F-L2 ND2***
						*15484*	*syn CYTB*
						***15740***	***L-F CYTb***

The table lists all the individual-specific coding region polymorphisms found in the sequences of the mtDNAs of cybrids TF3A5, E8, T8, F8, and TFA7. The first column in each doublet indicates the nucleotide position of the polymorphism, the second column indicates the gene name and the nucleotide change (in the 16S or 12S rRNA genes) or the amino acid change or syn for synonymous substitution (in the protein coding genes). Polymorphisms that cause an amino acid substitution are in boldface. New, previously unreported polymorphisms are indicated in italics. We searched two databases (http://www.phylotree.org [2];http://www.mitomap.org/MITOMAP [23]) and found these polymorphisms absent.

## Discussion

Since the report in 2003 [1] identifying the C150T transition as a longevity marker, several hypotheses have been proposed for explaining the advantage of the C150T transition. The C150T transition alters the location of H-strand replication origin. This may affect regulation of replication and in particular, may relax copy number control, allowing more mtDNA to be synthesized [1]. Although we have found no marked difference in the level of mtDNA between several cybrids of the U3a (C150T) haplogroup and cybrids of the J1c (150C) haplogroup (data not shown), there remains the possibility of a small difference in mtDNA level. Another possibility could be that the C150T transition could enhance the immune system in some way, perhaps by slowing the turnover of memory T-cells [24]. It is also possible that it is not the C150T *per se* that confers longevity, but rather one or more of the other polymorphisms that occur together with the C150T in a particular haplogroup.

We have carried out a biochemical and molecular analysis of cybrids of four different haplogroups. We measured doubling time, respiration rate, mitochondrial protein synthesis rate, membrane potential, and reactive oxygen species (ROS) production rate. The U3a and the J2b cybrids, which carry the C150T transition, exhibited a statistically significant lower ROS production rate than their haplogroup-matched correlates, which do not carry that transition. Haplogroup U and subhaplogroup J2 have been associated with longevity in population studies [3], [4]. No other parameter we measured showed a correlation with the presence of T at position 150 or with the longevity haplogroups. Thus, the lower ROS production rate is due to the T at position 150, and/or to one or more of the polymorphisms defining the haplogroup or specific to the individual.

Cybrids with haplogroup U-K2, which has C at position 150, and which has also been associated with longevity, have a lower ROS production rate, on average, than the cybrids of haplogroup J1c, but the difference is not statistically significant (P = 0.1885). Haplogroup J1 has a negative correlation with longevity [4]. Thus the U-K2-specific mtDNA polymorphisms may confer an advantage for longevity by some means other than by decreasing ROS production.

ROS generation occurs by the leakage of electrons from the respiratory chain directly to O_2_. Thus, one might expect that a higher respiration rate would be associated with a higher ROS production rate. The respiration rates differed markedly among the cybrid lines, with the U3a cybrids having the highest respiration rates. A high respiration rate associated with U haplogroup had been observed previously [12]. Contrary to expectation, the U3a cybrids had the lowest ROS production rates among the cybrids tested.

Our sequence analysis of cybrid mtDNA confirmed and refined the haplogroup assignment of the cybrid groups (Tables 2, 3). The U cybrid mtDNA sequence revealed that the donor's subgroup was U3a and that the donor's mtDNA sequence was nearly identical to two sequences recently added to the database [25] and identified as U3a1c [2]. The mtDNA coding region polymorphisms in this cybrid were limited almost exclusively to U- and U3a-specific polymorphisms (Tables 3,4). There were no individual-specific polymorphisms in the protein-coding genes except for three sites of heteroplasmy (Table 4). Thus, if the three heteroplasmies, which occur at about 50%, are neutral, it is possible that the U3a-specific threonine to alanine substitution in ND4L and/or the U3a-specific threonine to methionine substitution in ND5 (Table 3) are important for lowered ROS production and high respiration rates. Alternatively, polymorphisms associated with the U-K2 and J1c haplogroups may be responsible for greater ROS production rates.

A polymorphism that occurs in both 150C-containing haplogroups, which may be responsible for increased ROS production rates, occurs in the cytochrome b gene. The base change at 14798 causes a leucine for phenylalanine substitution and is present in both the U-K and J1c haplogroups. The structure of bovine complex III, including that of cytochrome b has been solved [26] and the amino acids in the following discussion are conserved between cow and man. The 14798 polymorphism changes the 18^th^ amino acid in cytochrome b from phenylalanine to leucine. The phenylalanine occurs at the end of a very short helix near the NH_2_-terminal end of the protein, at the inner coenzyme Q_10_ (CoQ)-binding site (Q_i_). It interacts with three of the carbons of ubiquinone [27]. Leucine in the place of phenylanaline would likely interact less well with CoQ and thus might destabilize CoQ binding and thereby inhibit the reduction of CoQ at the Q_i_ site. The inhibition of the Q cycle would increase the steady state concentration of the radical semiquinone at the outer coenzyme Q_10_ (CoQ)-binding site (Q_o_), which would lead to ROS generation.

The J2 haplogroup has another cytochrome b non-synonymous cytochrome b mutation, namely that at 15257. This base change substitutes an asparagine for aspartate at the 171^st^ amino acid position. This is near the Rieske iron-sulfur subunit in the complex and may be involved in stabilizing the interaction of cytochrome b with the iron-sulfur protein [28]. An amino acid substitution here could affect the rate of oxidation of CoQ at Q_o_ [29]. On the other hand, Fig. 4 shows that complex III-dependent respiration rates are not specifically lower in the J2 cybrids. The J2b-specific polymorphism at 15812 causes a methionine for valine substitution at amino acid 356. The valine is not conserved between phyla and is not directly involved in binding ubiquinone or other ligands of the complex [29]. Thus this polymorphism is likely to have no effect on the function of the complex.

Regarding the U-K2 cybrids, there are non-synonymous protein coding polymorphisms besides the one at 14798 that might account for its phenotype differences from the U3a cybrids. These are in the genes for ATP synthase subunit 6 (UK-specific) and ND2 (U-K2a-specific).

Also in the J1c cybrids there are individual-specific non-synonymous changes in the genes for ND2 and cytochrome b. The polymorphism at 15740 causes a phenylalanine for leucine substitution at the 332^nd^ amino acid in cytochrome b. This amino acid occurs in a helix that is in close proximity to another helix. The somewhat bulkier phenylalanine may cause a displacement of one or both helices to accommodate the phenylalanine. However, this amino acid is not involved in binding substrate or ligand, thus, the substitution of leucine with phenylalanine may not have any effect.

There were also Control Region polymorphisms: some were haplotype-specific, and some, individual specific (Table 2). In the U and U-K cybrids, the individual-specific polymorphisms were in either Hypervariable segment 1 (C16301T and T16356C, present in the U3a individual) or in Hypervariable segment 2 (C64T, present in the U-K2 individual). Nucleotides 16301 and 16356 are located inside the D-loop region, i.e. the portion of mtDNA that is frequently replicated in an abortive manner to yield 7S DNA [30], [31]. The C16301T and T16356C polymorphisms have been observed several times, as individual-specific polymorphisms, within several haplogroups, including M, N, and D [2]. The C64T polymorphism is ∼100 base pairs downstream of the two or three most commonly used origins of H-strand replication [32], [33] and 7 base pairs upstream of the origin that is used by cells recovering from mtDNA depletion [31]. The C64T polymorphism has been reported many times [23]. This polymorphism has been observed also in several haplogroup backgrounds, as a branch-specifying polymorphism (in haplogroups A2 and R0) or as an individual-specific polymorphism (in haplogroups F1, H1, H57 and K1) [2]. It is conceivable that this polymorphism, C64T, or the other two Control Region polymorphisms, C16301T and T16356C, could affect mtDNA replication and alter the mtDNA copy level. However, a change in mtDNA level would be expected to result in a change in protein synthesis rates, but Figure 2 shows that protein synthesis rates in the U3a and the U-K2 cybrids are not different at a statistically significant level.

The J2b and J1c cybrids also differ in the Control Region at several nucleotide positions. Two of them, C462T (J1-specific) and T482C (J1c1-specific) are not far from the L-strand promoter and thus could conceivably affect the production of transcripts of the L-strand, or, because L-strand transcription plays a role in mtDNA synthesis, DNA replication could be affected. However we could not detect statistically significant differences in protein synthesis rates (Fig. 2), suggesting that transcript levels are not significantly different between cybrids of the two different haplogroups.

Another Control Region polymorphism occurs in both the J1c and J2b cybrids, namely the C295T transition. This polymorphism occurs in one of the two binding site for the mitochondrial transcription factor, Tfam, that occur downstream of the L-strand promoter. Suissa and colleagues [13] have demonstrated that a DNA fragment that contains T at that position binds Tfam more tightly and is transcribed at a greater rate *in vitro* than the control DNA. Furthermore, they found that cybrids carrying the J haplogroup had substantially higher mtDNA levels than (but similar RNA levels as) cybrids carrying the H haplogroup (which has C at position 295). One possible interpretation of these data is that in the J cybrids, formation of DNA replication primers from nascent L-strand transcripts was enhanced and led to more mtDNA replication. We have compared mtDNA levels between several U3a cybrids (295C) and J1c cybrids (295T) and found no significant difference. This discrepancy may be explained by the presence of modifying Control Region polymorphisms occurring in our J or U cybrids or in the authors' H cybrids.

In conclusion, our experiments identify biochemical phenotypes that may help explain how haplogroup-associated polymorphisms determine longevity. We have discussed several haplogroup-specific polymorphisms and their possible effects on respiration and ROS production. A final determination of the effect of single polymorphisms awaits the development of methods that will allow us to change a single nucleotide in the mtDNA at will.

## Supporting Information

Table S1 MtDNA Sequencing Primers.
(DOCX)
