# Supplementary material for: Decreased Reactive Oxygen Species Production in Cells with Mitochondrial Haplogroups Associated with Longevity
Source: PLoS One. 2012 Oct 29;7(10):e46473. doi: 10.1371/journal.pone.0046473 (PMC3483264; doi:10.1371/journal.pone.0046473)
Supplement: Table S1 — MtDNA Sequencing Primers. (DOCX) [file pone.0046473.s001.docx]

Supplementary Table

MtDNA Sequencing Primers

| Oligo name | sequence | 5'pos. | 3'pos. |
| --- | --- | --- | --- |
| M13FL592 | TGTAAAACGACGGCCAGTCTCCTCAAAGCAATACACTG | 592 | 611 |
| M13RH2026 | CAGGAAACAGCTATGACCTGGACAACCAGCTATCACCA | 2026 | 2007 |
|  |  |  |  |
| M13FL1226 | TGTAAAACGACGGCCAGTCGATCAACCTCACCACCTCT | 1226 | 1245 |
| M13RH2026 | CAGGAAACAGCTATGACCTGGACAACCAGCTATCACCA | 2026 | 2007 |
|  |  |  |  |
| M13FL1830 | TGTAAAACGACGGCCAGTGGACTAACCCCTATACCTTCTGC | 1830 | 1852 |
| M13RH3365 | CAGGAAACAGCTATGACCAGGAATGCCATTGCGATTAG | 3365 | 3346 |
|  |  |  |  |
| M13FL3150 | TGTAAAACGACGGCCAGTTACTTCACAAAGCGCCTTCC | 3150 | 3169 |
| M13RH4679 | CAGGAAACAGCTATGACCAAGGATTATGGATGCGGTTG | 4679 | 4660 |
|  |  |  |  |
| M13FL3777 | TGTAAAACGACGGCCAGTTGGCTCCTTTAACCTCTCCA | 3777 | 3796 |
| M13RH4679 | CAGGAAACAGCTATGACCAAGGATTATGGATGCGGTTG | 4679 | 4660 |
|  |  |  |  |
| M13FL4466 | TGTAAAACGACGGCCAGTACTAATTAATCCCCTGGCCC | 4466 | 4485 |
| M13RH6050 | CAGGAAACAGCTATGACCACCTAGAAGGTTGCCTGGCT | 6050 | 6031 |
|  |  |  |  |
| M13FL5238 | TGTAAAACGACGGCCAGTCTAACCGGCTTTTTGCCC | 5238 | 5255 |
| M13RH6050 | CAGGAAACAGCTATGACCACCTAGAAGGTTGCCTGGCT | 6050 | 6031 |
|  |  |  |  |
| M13FL5835 | TGTAAAACGACGGCCAGTGAGGCCTAACCCCTGTCTTT | 5835 | 5854 |
| M13RH7334 | CAGGAAACAGCTATGACCAGCGAAGGCTTCTCAAATCA | 7334 | 7315 |
|  |  |  |  |
| M13FL7129 | TGTAAAACGACGGCCAGTACGCCAAAATCCATTTCACT | 7129 | 7148 |
| M13RH8816 | CAGGAAACAGCTATGACCTGGGTGGTTGGTGTAAATGA | 8816 | 8797 |
|  |  |  |  |
| M13FL7908 | TGTAAAACGACGGCCAGTACGAGTACACCGACTACGGC | 7908 | 7927 |
| M13RH8816 | CAGGAAACAGCTATGACCTGGGTGGTTGGTGTAAATGA | 8816 | 8797 |
|  |  |  |  |
| M13FL8602 | TGTAAAACGACGGCCAGTTTTCCCCCTCTATTGATCCC | 8602 | 8621 |
| M13RH10149 | CAGGAAACAGCTATGACCTGTAGCCGTTGAGTTGTGGT | 10149 | 10130 |
|  |  |  |  |
| M13FL9967 | TGTAAAACGACGGCCAGTTCTCCATCTATTGATGAGGGTCT | 9967 | 9989 |
| M13RH11511 | CAGGAAACAGCTATGACCTTGAGAATGAGTGTGAGGCG | 11511 | 11492 |
|  |  |  |  |
| M13FL10653 | TGTAAAACGACGGCCAGTGCCATACTAGTCTTTGCCGC | 10653 | 10672 |
| M13RH11511 | CAGGAAACAGCTATGACCTTGAGAATGAGTGTGAGGCG | 11511 | 11492 |
|  |  |  |  |
| M13FL11295 | TGTAAAACGACGGCCAGTTCACTCTCACTGCCCAAGAA | 11295 | 11314 |
| M13RH12793 | CAGGAAACAGCTATGACCAGAAGGATATAATTCCTACG | 12793 | 12774 |
|  |  |  |  |
| M13FL11929 | TGTAAAACGACGGCCAGTTATCACTCTCCTACTTACAG | 11929 | 11948 |
| M13RH12793 | CAGGAAACAGCTATGACCAGAAGGATATAATTCCTACG | 12793 | 12774 |
|  |  |  |  |
| M13FL12546 | TGTAAAACGACGGCCAGTAACCCAAACAACCCAGCTCT | 12546 | 12565 |
| M13RH13995 | CAGGAAACAGCTATGACCTAGGAGGAGTAGGGGCAGGT | 13995 | 13976 |
|  |  |  |  |
| M13FL13319 | TGTAAAACGACGGCCAGTACATCTGTACCCACGCCTTC | 13319 | 13338 |
| M13RH13995 | CAGGAAACAGCTATGACCTAGGAGGAGTAGGGGCAGGT | 13995 | 13976 |
|  |  |  |  |
| M13FL13805 | TGTAAAACGACGGCCAGTCCCTCGCTGTCACTTTCCTAG | 13805 | 13825 |
| M13RH15178 | CAGGAAACAGCTATGACCTGTGGCCCCTCAGAATGATA | 15178 | 15159 |
|  |  |  |  |
| M13FL14977 | TGTAAAACGACGGCCAGTAATCATCCGCTACCTTCACG | 14977 | 14996 |
| M13RH16439 | CAGGAAACAGCTATGACCGCACTCTTGTGCGGGATATT | 16439 | 16420 |
|  |  |  |  |
| M13FL15759 | TGTAAAACGACGGCCAGTTCGGAGGACAACCAGTAAGC | 15759 | 15778 |
| M13RH16439 | CAGGAAACAGCTATGACCGCACTCTTGTGCGGGATATT | 16439 | 16420 |
|  |  |  |  |
| M13FL16102 | TGTAAAACGACGGCCAGTTACTGCCAGCCACCATGAA | 16102 | 16120 |
| M13RH794 | CAGGAAACAGCTATGACCAGGCTAAGCGTTTTGAGCTG | 794 | 775 |
|  |  |  |  |
| M13FL369 | TGTAAAACGACGGCCAGTCCCTAACACCAGCCTAACCA | 369 | 388 |
| M13RH794 | CAGGAAACAGCTATGACCAGGCTAAGCGTTTTGAGCTG | 794 | 775 |
